# Anaphylactic reaction to intravenous corticosteroids in the treatment of ocular toxoplasmosis: a case report

**DOI:** 10.1186/1752-1947-8-110

**Published:** 2014-04-02

**Authors:** Achim Fieß, Sven Halstenberg, Antonia Fellas, Inez Frisch, Ulrich Helmut Steinhorst

**Affiliations:** 1 Department of Ophthalmology, Dr. Horst-Schmidt-Clinics, Wiesbaden, Germany; 2 Department of Dermatology, Dr. Horst-Schmidt-Clinics, Wiesbaden, Germany; 3 Center of Ophthalmology, Ingelheim, Germany

**Keywords:** Anaphylactic reaction, Corticosteroids, Ocular toxoplasmosis, Skin-prick test, Treatment

## Abstract

**Introduction:**

This case report presents for the first time an acute systemic allergic reaction to corticosteroids in a patient with ocular toxoplasmosis after treatment with intravenous cortisone, and discusses alternative treatments.

**Case presentation:**

We present the case of a 57-year-old Caucasian woman with an anaphylactic reaction after intravenous injection of prednisolone-21-hydrogensuccinate (Solu-Decortin® H) given for the treatment of toxoplasmosis-associated chorioretinitis. Immediately after the injection, she developed an acute erythema of the legs and abdomen, angioedema, hypotension (blood pressure 80/40mmHg), tachycardia (heart rate 140/minute), hyperthermia (38.8°C), and respiratory distress. Allergological examinations showed a positive skin-prick test to prednisolone and methylprednisolone. In addition, an oral exposure test with dexamethasone (Fortecortin®) and betamethasone (Celestamine®) was conducted to find alternative corticosteroids for future treatments. After oral application, no local or systemic reactions were observed for these two substances.

**Conclusions:**

This case report demonstrates that systemic allergic reactions are possible in patients with uveitis or other inflammatory ophthalmological conditions treated with intravenous corticosteroids. Intravenous administration of cortisone, for example, in the treatment of ocular toxoplasmosis, should always be conducted with caution because of a possible allergic reaction. For patients who react to a particular steroid, it is necessary to undergo allergological testing to confirm that the compound in question is indeed allergenic, and to identify other corticosteroids that are safe for future anti-inflammatory treatments.

## Introduction

Glucocorticoids are among the most effective and longest known pharmaceuticals for the suppression of immunological reactions. Since their initial therapeutic use in medicine in 1948 [1], they have been used for their anti-inflammatory effect in a variety of indications. There are also a lot of indications in ophthalmology, for example, treatment of uveitis or ocular toxoplasmosis (OT). However, since their first use, many intolerance reactions have been described. These were mainly reported for local [2], intramuscular [3], oral [4], and inhalation [5] applications. Only a few cases report an anaphylactic reaction after intravenous administration of cortisone [6,7]. Therefore, we report for the first time the case of a systemic allergic reaction by a patient with OT treated with a single dose of intravenous prednisolone-21-hydrogensuccinate (Solu-Decortin® H) to reduce the ocular inflammatory response of OT.

## Case presentation

**Figure 1:**
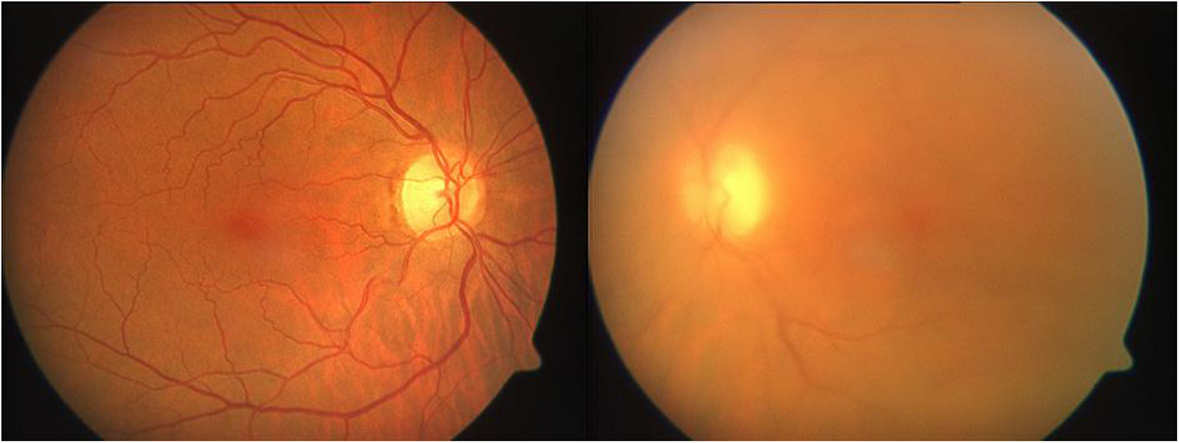
Fundus examination of the left eye revealed vitreous opacities and chorioretinal infiltrates.

A 57-year-old Caucasian woman reported a decrease in visual acuity for 2 weeks. Her best-corrected visual acuity for her right eye was 20/20 and for her left eye 20/100. A slit-lamp examination and intraocular pressure were unremarkable in both eyes. Fundus examination of her left eye revealed temporal intraretinal infiltrates and vitreous opacities (Figure 1). Her right eye was unremarkable.

**Figure 2:**
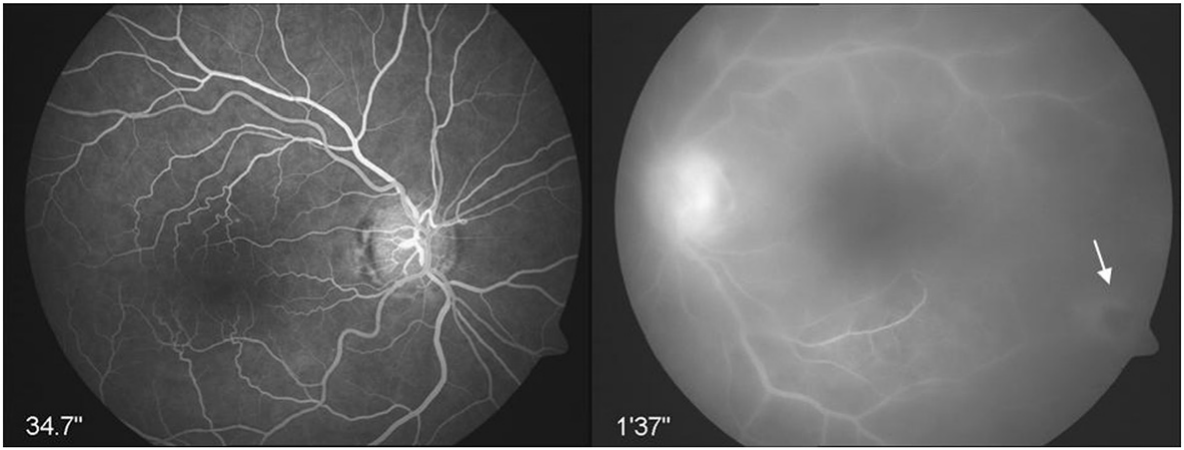
Fluorescein angiography of the left eye revealed vitreous opacities, a hyperfluorescence of the optic disc, leakage along the vessels, and chorioretinal infiltrates.

Fluorescein angiography showed hyperfluorescence of her optic disc, leakage along the vessels, and chorioretinal hyperfluorescent infiltrates (Figure 2). The diagnosis of OT was based on the typical morphology of her ocular lesions and a positive serological testing for *Toxoplasma gondii* (immunoglobulin G concentration = 537IU/mL).

**Figure 3:**
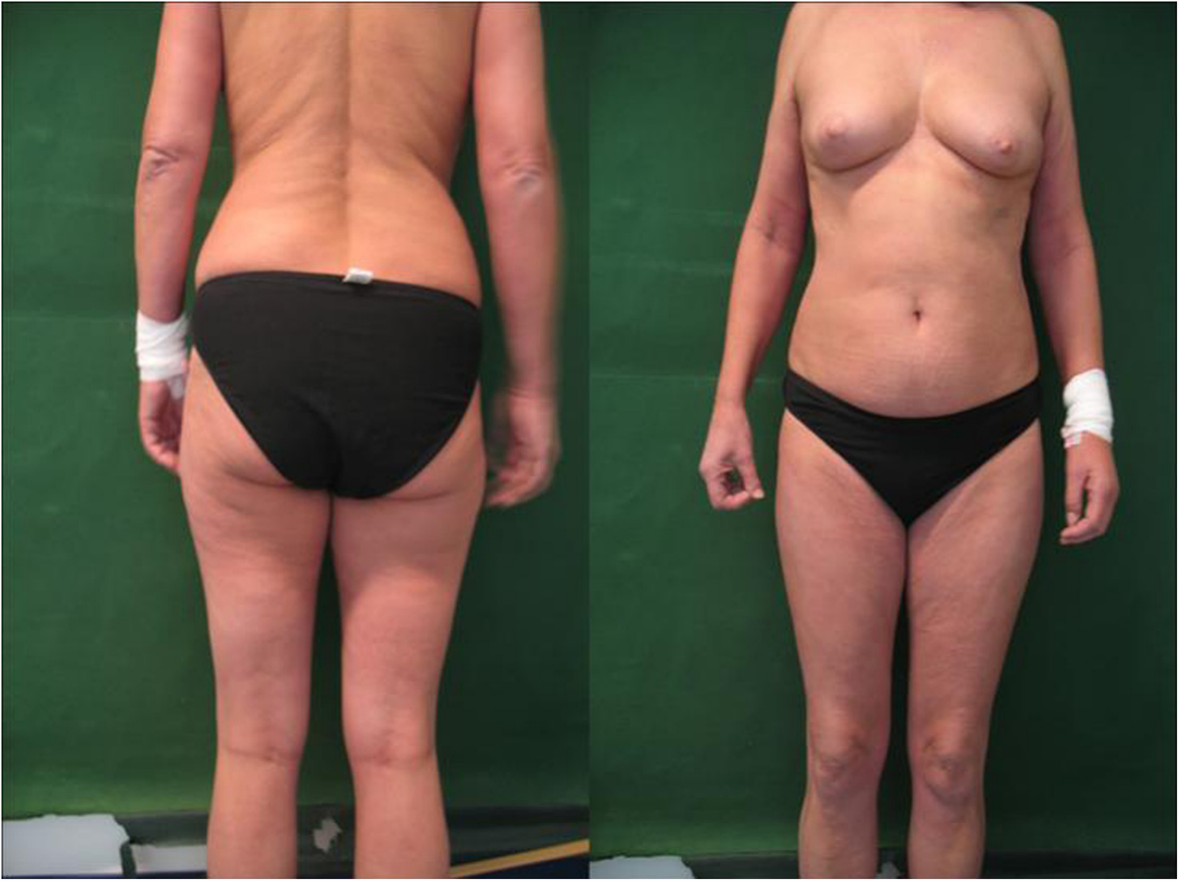
Erythema, particularly of the legs and abdomen, and angioedema after intravenous injection of prednisolone (photo was taken after return from Intensive Care Unit).

She was treated with clindamycin (Clindamycin® H) 4 × 300mg daily over 3 days without any significant improvement of visual acuity. On the fourth day of hospitalization, she received 100mg prednisolone-21-hydrogensuccinate (Solu-Decortin® H) intravenously in addition to her treatment with clindamycin (Clindamycin® H). Within 2 minutes she developed an acute erythema, particularly of her legs and abdomen (Figure 3), angioedema, hypotension (blood pressure 80/40mmHg), tachycardia (heart rate 140/minute), hyperthermia (38.8°C), and respiratory distress. Subsequently, she was transferred to the Intensive Care Unit to be monitored and treated with clemastine fumarate (Tavegil®), ranitidin (Ranitic®), and intravenous fluids. After 1 hour she recovered and after 12 hours she was transferred back to the ophthalmological ward. Her erythema and angioedema persisted for 32 hours. She had no history of previous steroid use. Subsequent allergy testing was conducted after 3 months in the Department of Dermatology in our hospital. The testing showed a positive skin-prick test for prednisolone and methylprednisolone in the form of a 5mm wheal, and negative results for dexamethasone and hydrocortisone (Table 1), which confirmed her suspected allergy to prednisolone. Because of her allergic reaction to class A (prednisone-type) corticosteroids and possible complications due to cross-reactions to class D2 (prednicarbate-type) corticosteroids, we avoided any further treatment with systemic or intravitreal corticosteroids.

**Table 1:** Skin-prick test

**Corticosteroids**	**Skin-prick test 24 hours**
Hydrocortisone	–
Triamcinolone acetonide	–
Clobetasol-17-propionate	–
Hydrocortisone-17-butyrate	–
Betamethasone-17-valerate	–
Dexamethasone	–
Budesonide	–
Prednisolone	+
Methylprednisolone	+
Fluocinonide	–
Hydrocortisone acetate	–
Diflucortolone	–
Fluocortolon	–
Histamine	++
0.9% Sodium chloride	–

Positive (+) tests for prednisolone and methylprednisolone in the skin-prick test. The other compounds tested produced negative (-) test results in the skin-prick test and all compounds showed negative results in the epicutaneous test.

In addition, an oral exposure test with a step-by-step elevation of doses up to 2.0mg of dexamethasone (Fortecortin®) and 1.5mg betamethasone (Celestamine®) was conducted to find alternative corticosteroids for future treatments. For both substances, no local or systemic side effects were observed.

After 1 month of treatment with clindamycin (Clindamycin® H) monotherapy without any increase in visual acuity, the patient underwent a vitrectomy with balanced salt solution filling because of heavy vitreous opacities, and to obtain a sample of the vitreous body. The polymerase chain reaction (PCR) on the sample was positive for *Toxoplasma gondii*.

**Figure 4:**
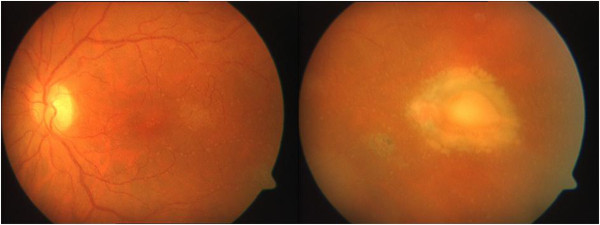
Fundus examination of the left eye after vitrectomy revealed a clearer insight, and chorioretinal scars.

**Figure 5:**
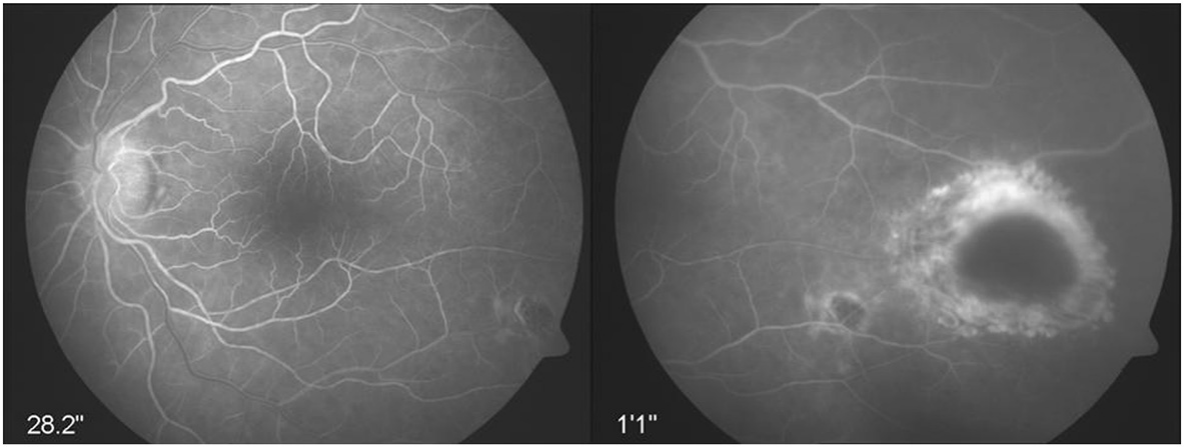
Left eye showing chorioretinal infiltrates and a temporal scar as detected by fluorescein angiography.

The postoperative examination of her left eye revealed chorioretinal scars and infiltrates (Figure 4). Her right eye was still unremarkable. Fluorescein angiography of her left eye also revealed hyperfluorescent intraretinal infiltrates of the temporal hemisphere and a scar of the inferotemporal retinal vein branch (Figure 5). Her visual acuity recovered to right eye 20/20 and left eye 20/40.

## Discussion

The first unquestionable anaphylactic reaction to intravenous corticosteroids was described in the year 1974 after treatment of a patient with methylprednisolone and hydrocortisone during an acute asthma attack [6]. The medical literature describes only approximately 40 cases of allergic reactions after systemic administration of cortisone [8,9]. Moreover, this is the first reported case of an acute allergic reaction to intravenous prednisolone in a patient with OT. Accordingly, this case demonstrates that the use of intravenous cortisone requires caution because of a possible allergic reaction, albeit very rare. In the case of a severe systemic reaction to intravenous corticosteroids, we advise allergological testing of the most frequently administered corticosteroids and of all other components of the administered formulation. This is, on the one hand, to find the compound responsible for the allergic reaction and, on the other hand, to identify corticosteroids for future treatment.

A few publications report a dramatic clinical course with subsequent death of the patient after intravenous administration of corticosteroids. However, in these cases the correlation between intravenous corticosteroids and subsequent death was hypothesized only due to the chronological connection [10,11]. Our patient showed the typical signs of anaphylaxis as described in a few publications and recovered completely after 2 days [12]. However, the hyperthermia of the patient after the cortisone administration is not a typical feature of anaphylaxis. Nevertheless, since there was no concurrent infection other than OT, it was concluded that hyperthermia was part of the anaphylactic reaction in this case, although it is not commonly so.

In the literature, there is epidemiological data about the incidence of corticosteroid allergies. In a routine epicutaneous test in 2073 patients there was a positive test result in 61 patients (2.9%) [13]. In a study by Alexiou *et al.*[12], in three of 300 patients an anaphylactic reaction to prednisolone-21-hydrogensuccinate (Solu-Decortin® H) occurred during a 3-day therapy with intravenous cortisone up to 1000mg per day. This would correspond to a frequency of 1%. In contrast to this study, the number of case reports in the medical literature is very low [8,9].

To confirm the suspected allergy to corticosteroids in the present case, a skin-prick test and an epicutaneous test were carried out. The skin-prick test was performed by puncturing the skin of the patient’s lower arm with a lancet in an area where a drop of the possible allergenic solution had been applied. Immediately after puncturing the antigen solution was wiped off and following 5 to 15 minutes and 24 hours, the skin reaction was evaluated. A positive reaction was noted if there was a wheal at the puncture. There was a positive control (histamine application) and a negative control (0.9% sodium chloride application). In the present case, the skin-prick test was positive in the form of a 5mm wheal for prednisolone and methylprednisolone after 24 hours. Due to the fact that the skin-prick test was positive for pure preparations of corticosteroids, there was no need to test for other candidate substances. Solu-Decortin® H is sealed in glass ampules under nitrogen, which can hardly be a cause for an allergy. If there had been additional ingredients such as preservatives, these should have been tested as well. It is important to conduct allergological testing for every suspected substance because every compound administered could indeed be allergenic. Given the positive prick-test an intracutaneous test was waived because of the danger of a possible anaphylactic reaction. The epicutaneous test of our patient was negative for all substances being tested. This could be because an epicutaneous test is a test for Type IV reactions. The epicutaneous test was performed to test if the patient had a possible contact allergy for corticosteroids. However, the anaphylactic reaction in our case was a Type I reaction, which could be confirmed with the skin-prick test that tests Type I reactions. Overall, immunological reactions to systemic corticosteroids are frequently of Type I according to the classification by Gell and Coombs [14].

Detection of antibodies is often difficult because there are no specific data for the technical analysis of serum samples. Specific serum IgE antibodies to corticosteroids were detected in only a few cases [15-17] and often antibodies could not be found [12].

The clinical course of OT is usually self-limiting but can recur after years. However, in some cases, OT can lead to loss of vision, optic nerve or macular affection, and vitreous opacities [18]. Previous studies [19] have shown that pars plana vitrectomy can be performed safely on patients with OT who suffer from vitreoretinal complications. Our findings in this case report are consistent with these results. In the case of our patient, after the vitrectomy, her visual acuity recovered due to the removal of vitreous opacities. Moreover, another benefit is the diagnostic certainty obtained by conducting PCR analysis on a sample of vitreous body when a diagnosis remains inconclusive solely based on clinical symptoms.

The most common therapy for OT is a triple therapy consisting of pyrimethamine, sulfadiazine, and corticosteroids [20]. Steroids are successful in reducing the ocular inflammatory response. To reduce ocular inflammation, the use of intravitreal triamcinolone or dexamethasone is an alternative treatment for patients with OT. A study by Soheilian *et al.*[21] compared intravitreal injection of clindamycin and dexamethasone with the standard treatment for OT, consisting of systemic pyrimethamine, sulfadiazine, and prednisolone. The authors showed that intravitreal injections of clindamycin and dexamethasone resulted in lower systemic side-effects and greater convenience than systemic OT medications. However, in the case of a proven prednisolone allergy, intravitreal injection of an alternative steroid, for example dexamethasone, would only be feasible if an allergic reaction towards the intravitreal deposit of the drug can definitely be excluded. Due to the possibility of an allergic cross-reaction toward dexamethasone, which would probably reside in the eye for weeks after intravitreal deposition, we strictly avoided such a treatment.

## Conclusions

In summary, this case report demonstrates that systemic allergic reactions are possible in patients with uveitis or other inflammatory ophthalmological conditions treated with intravenous corticosteroids. Intravenous administration of cortisone, for example in the treatment of OT, should always be conducted with caution because of a possible allergic reaction. For patients who react to a particular steroid, it is necessary to undergo allergological testing to confirm that the compound in question is indeed allergenic, and to identify other corticosteroids that are safe for future anti-inflammatory treatments.

## Consent

Written informed consent was obtained from the patient for publication of this case report and accompanying images. A copy of the written consent is available for review by the Editor-in-Chief of this journal.

## Competing interests

The authors declare that they have no competing interests.

## Authors’ contributions

AFi made substantial contributions to conception and design, acquisition of data, analysis and interpretation of data, and he has been involved in drafting the manuscript or revising it critically for important intellectual content. SH has made substantial contributions to the article, and he has been involved in drafting the manuscript and revising it critically. AFe has made substantial contributions to the article, and he has been involved in drafting the manuscript and revising it critically. IF has been involved in drafting the manuscript and revising it critically and has given final approval of the version to be published. UHS has been involved in drafting the manuscript and revising it critically and has given final approval of the version to be published. All authors read and approved the final manuscript.

## References

[B1] Hench PS Kendall EC The effect of a hormone of the adrenal cortex (17-hydroxy-11-dehydrocorticosterone; compound E) and of pituitary adrenocorticotropic hormone on rheumatoid arthritis Proc Staff Meet Mayo Clin 1949 24 8 181 197 18118071

[B2] Burckhardt W Kontaktekzem durch Hydrocortison Hautarzt 1959 10 42 43 13806005

[B3] Rodger RSC Anaphylaxis following treatment with a corticosteroid report of one case Clin Allergy 1983 13 499 500 10.1111/j.1365-2222.1983.tb02628.x 6627626

[B4] Rasanen L Hasan T Allergy to systemic and intralesional corticosteroids Br J Dermatol 1993 128 4 407 411 10.1111/j.1365-2133.1993.tb00200.x 8494754

[B5] Derrick EK Price ML Contact dermatitis to Pulmicort inhaler Br J Dermatol 1995 133 417669639

[B6] Mendelson LM Meltzer EO Hamburger RN Anaphylaxis-like reactions to corticosteroid therapy J Allergy Clin Immunol 1974 54 3 125 131 10.1016/0091-6749(74)90049-9 4850575

[B7] Atanasković-Marković M Gavrović-Jankulović M Janković S Blagojević G Cirković-Velicković T Milojević I Simić D Nestorović B Immediate allergic reaction to methylprednisolone with tolerance of other corticosteroids Srp Arh Celok Lek 2012 140 3–4 233 235 22650114

[B8] Uter W Allergische Reaktionen auf Glukokortikoide Dermatosen 1990 38 75 90 2142917

[B9] Saff DM Taylor JS Vidimos AT Allergic reaction to intralesional triamcinolone acetonide: a case report Arch Dermatol 1995 131 6 742 743 10.1001/archderm.1995.01690180122031 7778940

[B10] al Mahdy H Hall M Anaphylaxis and hydrocortisone Ann Intern Med 1988 108 3 487 488 10.7326/0003-4819-108-3-487_2 3341687

[B11] Thompson JF Chalmers DH Wood RF Kirkham SR Morris PJ Sudden death following high-dose intravenous methylprednisolone Transplantation 1983 36 5 594 596 10.1097/00007890-198311000-00028 6356526

[B12] Alexiou C Kau RJ Luppa P Arnold W Klinische Bedeutung allergischer Reaktionen bei Glukokortikoidbehandlung Laryngo-Rhino-Otol 1999 78 573 578 10.1055/s-1999-8755 10595343

[B13] Dooms-Gossens A Morren M Results of routine patch testing with corticosteroid series in 2073 patients Contact Dermatitis 1992 26 182 191 10.1111/j.1600-0536.1992.tb00290.x 1505184

[B14] Gell PGH Coombs RRA Coombs RRA, Gell PGH The classification of allergic reactions underlying disease Clinical Aspects of Immunology 1963 Blackwell Science

[B15] Rasanen L Tarvainen K Makinen-Kiljunen S Urticaria to hydrocortisone Allergy 2001 56 4 352 353 10.1034/j.1398-9995.2001.00056.x 11284808

[B16] Pryse-Phillips WE Chandra RK Rose B Anaphylactoid reaction to methylprednisolone pulsed therapy for multiple sclerosis Neurology 1984 34 8 1119 1121 10.1212/WNL.34.8.1119 6540393

[B17] Burgdorff T Venemalm L Vogt T Landthaler M Stolz W IgE-mediated anaphylactic reaction induced by succinate ester of methylprednisolone Ann Allergy Asthma Immunol 2002 89 4 425 428 10.1016/S1081-1206(10)62046-7 12392389

[B18] Holland GN Ocular toxoplasmosis: a global reassessment Part I: epidemiology and course of disease. Am J Ophthalmol 2003 136 6 973 988 10.1016/j.ajo.2003.09.040 14644206

[B19] Adan A Giralt J Alvarez G Alforja S Bures-Jesltrup A Casaroli-Marano RP Corcostegui B Pars plana vitrectomy for vitreoretinal complications of ocular toxoplasmosis Eur J Ophthalmol 2009 19 6 1039 1043 19882570 10.1177/112067210901900622

[B20] Montoya JG Liesenfeld O Toxoplasmosis Lancet 2004 363 9425 1965 1976 10.1016/S0140-6736(04)16412-X 15194258

[B21] Soheilian M Ramezani A Azimzadeh A Sadoughi MM Dehghan MH Shahghadami R Yaseri M Peyman GA Randomized trial of intravitreal clindamycin and dexamethasone versus pyrimethamine, sulfadiazine, and prednisolone in treatment of ocular toxoplasmosis Ophthalmology 2011 118 1 134 141 10.1016/j.ophtha.2010.04.020 20708269

